# Supplementary material for: Spatial release from masking in crocodilians
Source: Commun Biol. 2022 Aug 25;5:869. doi: 10.1038/s42003-022-03799-7 (PMC9411511; doi:10.1038/s42003-022-03799-7)
Supplement: Supplementary file 2 — Supplementary Information [file 42003_2022_3799_MOESM2_ESM.pdf]

Supplementary Information

Supplementary Figures

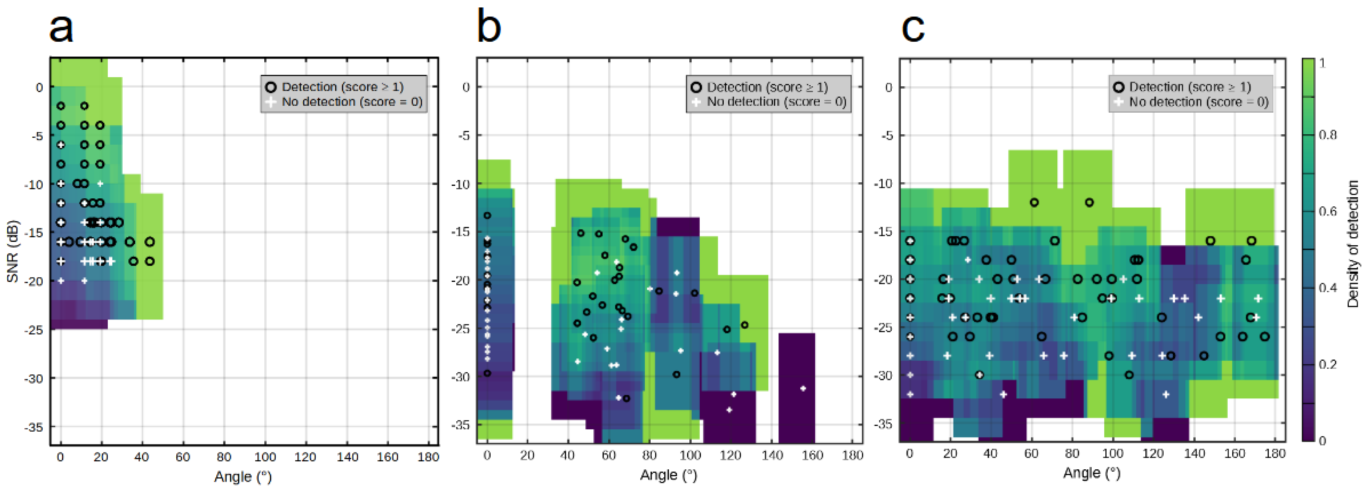

Supplementary Figure 1. **a** Ability of female caimans to detect a sound stimulus (young distress call) as a function of Signal-to-Noise Ratio (SNR) and loudspeakers spacing (Angle). Each performed trials (104 trials on 16 females) are symbolized are either a "o" (no reaction) or a "+" (at least a head or a body movement towards the target loudspeaker). The intensity of the behavioral reaction is not reported here. **b** Ability of young Nile crocodiles to detect a sound stimulus (contact call) as a function of Signal-to-Noise Ratio (SNR) and loudspeakers spacing (Angle). Each performed trials (71 trials on 8 individuals) are symbolized are either a "o" (no reaction) or a "+" (at least a head or a body movement towards the target loudspeaker). **c** Ability of young Nile crocodiles to detect a sound stimulus (synthetic buzz) as a function of Signal-to-Noise Ratio (SNR) and loudspeakers spacing (Angle). Each performed trials (120 trials on 2 individuals) are symbolized are either a "o" (no reaction) or a "+" (at least a head or a body movement towards the target loudspeaker). For **a**, **b** and **c**, the probability of detection is emphasized by the green-blue color scale, ranging from 0 (deep blue, no detection) to 1 (light green, effective detection; probabilities calculated using a sliding squared window 24° × 12 dB).

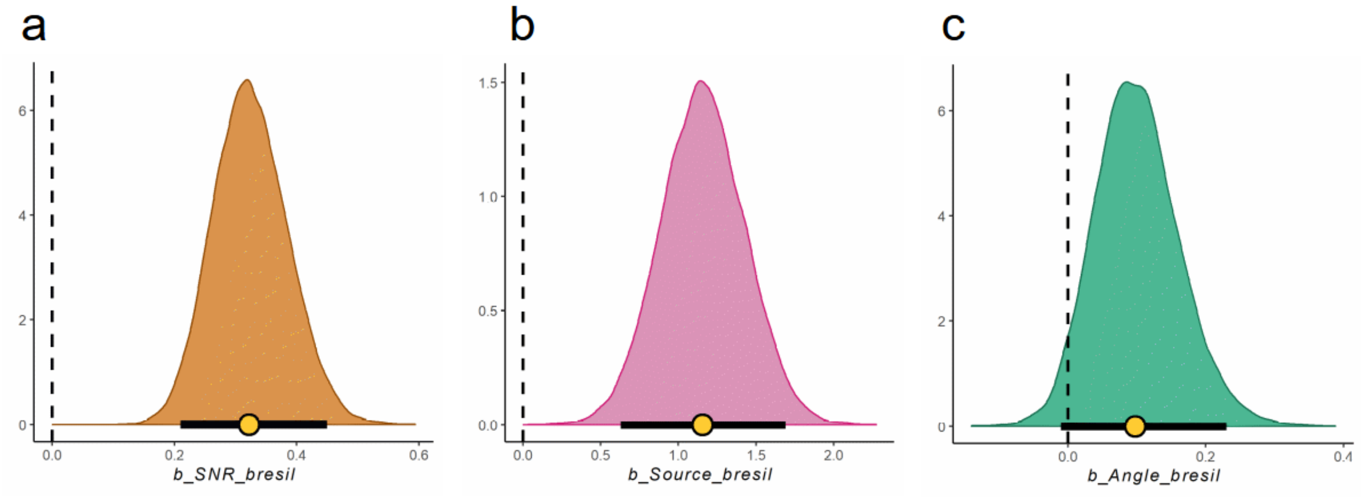

Supplementary Figure 2. Bayesian model outcome for the Experiment 1. Posterior density distributions for the effect of the SNR (**a**) and target position (**b**) on the behavioral score probability (see Supplementary Table 2). Posterior density distributions for the effect of separation angle (**c**) on the behavioral score probability (see Supplementary Table 5). Circles show posterior median distribution of the beta-coefficient with 95% credible intervals in black horizontal bars. Vertical dashed line represent a null effect, a positive effect size indicating an increased behavioral score/detection probability.

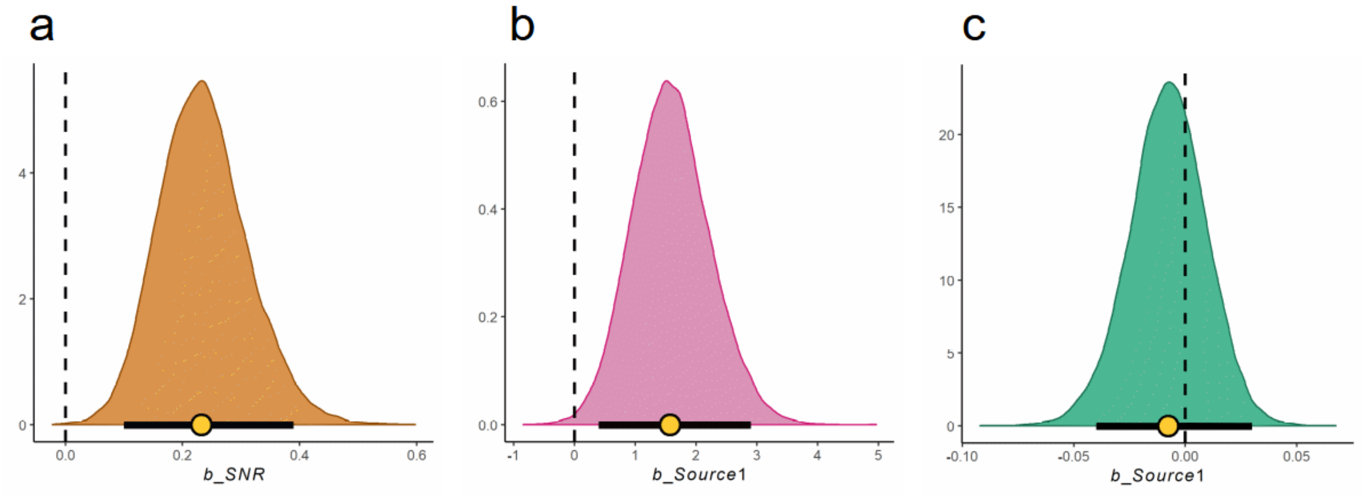

Supplementary Figure 3. Bayesian model outcome for the Experiment 2. Posterior density distributions for the effect of the SNR (**a**) and target position (**b**) on the detection probability (see Supplementary Table 7). Posterior density distributions for the effect of separation angle (**c**) on the detection probability (see Supplementary Table 9). Circles show posterior median distribution of the beta-coefficient with 95% credible intervals in black horizontal bars. Vertical dashed line represent a null effect, a positive effect size indicating an increased behavioral score/detection probability.

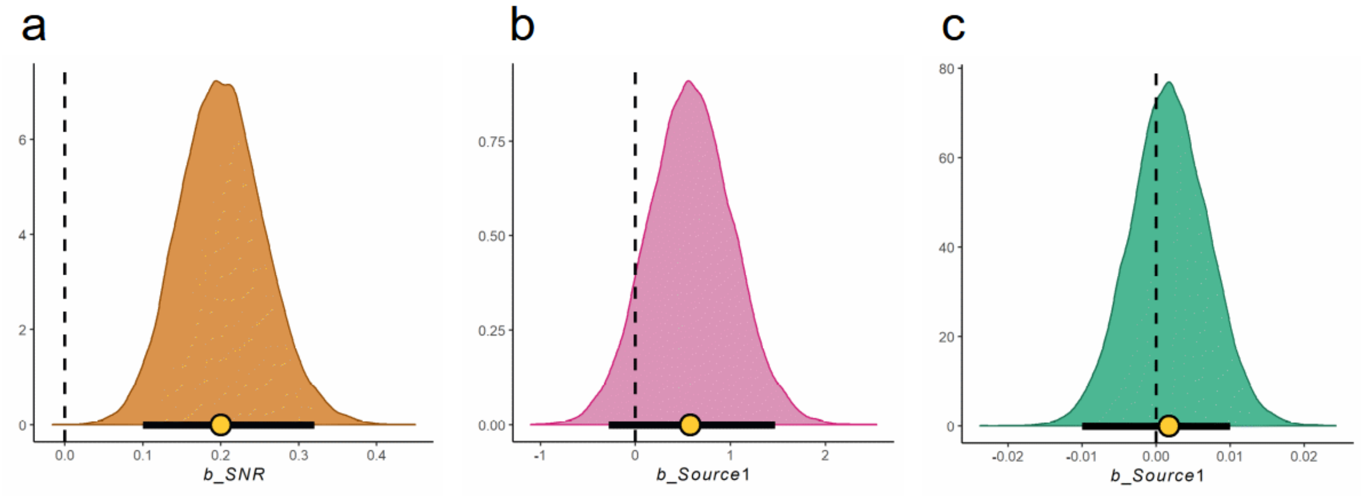

Supplementary Figure 4. Bayesian model outcome for the Experiment 3: posterior density distributions for the effect of the SNR (**a**) and target position (**b**) on the detection probability (see Supplementary Table 11). Posterior density distributions for the effect of separation angle (**c**) on the detection probability (see Supplementary Table 13).Circles show posterior median distribution of the beta-coefficient with 95% credible intervals in black horizontal bars. Vertical dashed line represent a null effect, a positive effect size indicating an increased behavioral score/detection probability.

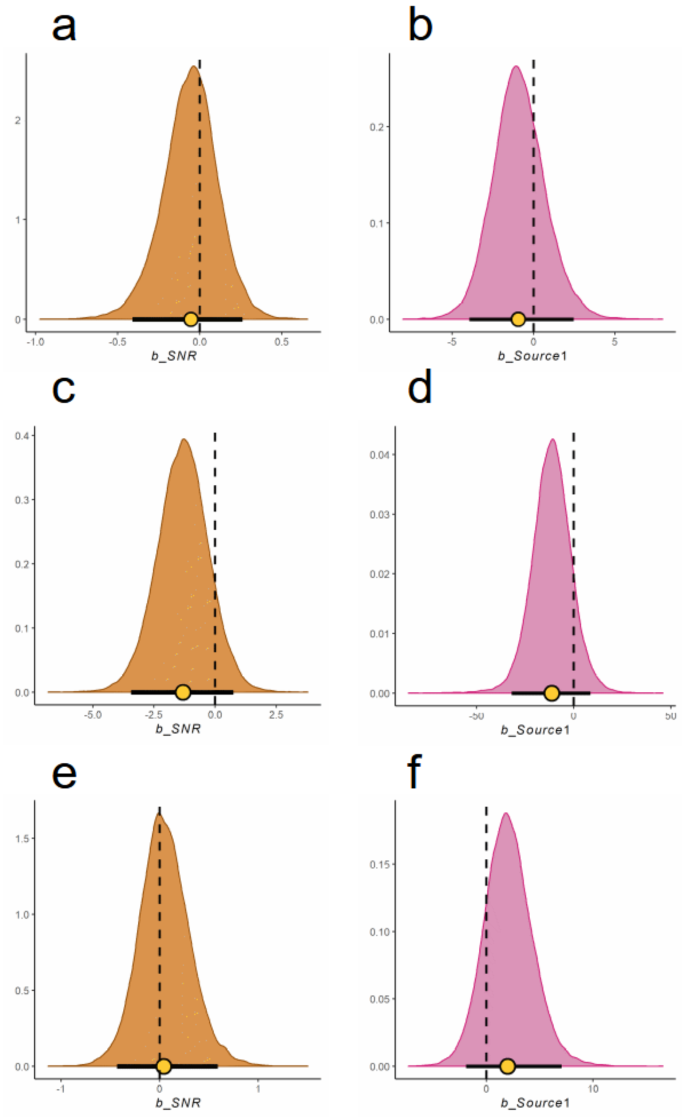

Supplementary Figure 5. Bayesian model outcome: posterior density distributions for the effect of the SNR (**a**, **c**, **e**) and target position (**b**, **d**, **f**) on the reaction time (see Supplementary Table 4). The panel **a** and **b** refers to the experiment 1, the panel **c** and **d** refers to the Experiment 2 and the panel **e** and **f** refers to the experiment 3. Circles show posterior median distribution of the beta-coefficient with 95% credible intervals in black horizontal bars. Vertical dashed line represent a null effect, a positive effect size indicating an increased behavioral score/detection probability.

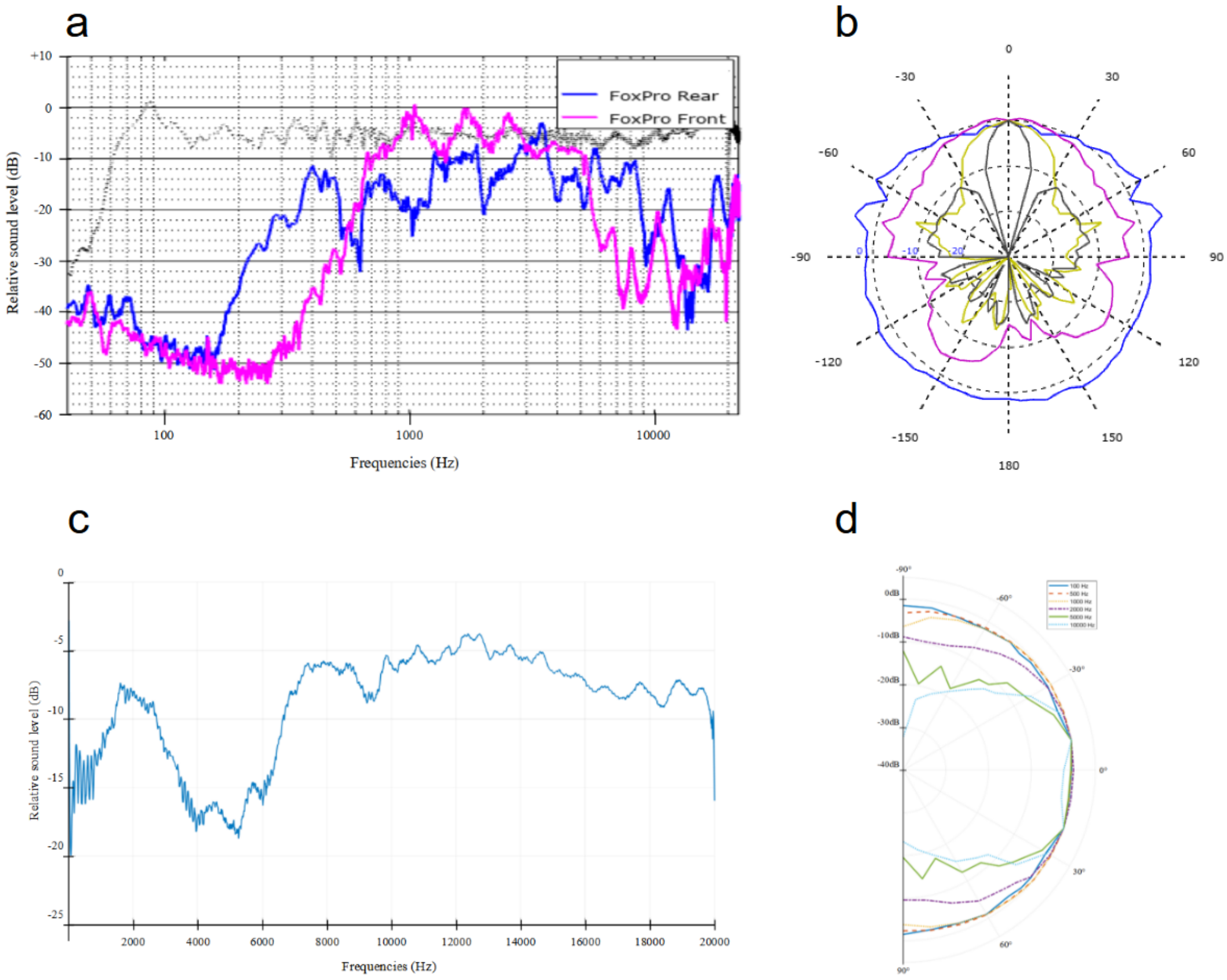

Supplementary Figure 6. **a** Frequency response of the FoxPro Fusion speakers (rear loudspeaker) used in Experiments 1 and 2. **b** Directivity pattern of the FoxPro Fusion speakers (rear loudspeaker) used in Experiments 1 and 2. **c** Frequency response of the AudioPro, Bravo Allroom Sat speakers used in Experiment 3. **d** Directivity pattern of the AudioPro, Bravo Allroom Sat speakers used in Experiment 3. All measurements were done in a semi-anechoic chamber with calibrated chains.

Supplementary Tables

**Tested female →<br>SNR (dB) ↓**	**1**	**2**	**3**	**4**	**5**	**6**	**7**	**8**	**9**	**10**	**11**	**12**	**13**	**14**	**15**	**16**
-20					×											
-18					×	×	×	×	×	×	×	×	×	×	×	×
-16			×	×	×	×	×	×	×	×	×	×	×		×	×
-14	×	×			×	×	×	×		×		×	×		×	×
-12					×		×			×			×			
-10					×		×			×			×			
-8							×			×						
-6							×			×						
-4							×			×						
-2							×									

Supplementary Table 1. Details of the trials performed during the experiment 1 (N = 16 female Jacare caimans). A cross "×" indicates that the tested female was challenged by both a co-located and separated stimulus at the corresponding SNR. An empty cell indicates that the female was neither challenged by a co-located nor a separated stimulus.

	Estimate	Est.Error	l-95% CI	u-95% CI
Intercept[0]	-4.75	1.03	-6.89	-2.86
Intercept[1]	-4.29	1.00	-6.37	-2.46
Intercept[2]	-3.35	0.95	-5.33	-1.62
Intercept[3]	-2.96	0.94	-4.93	-1.25
SNR	0.32	0.06	0.21	0.45
Target position	1.15	0.27	0.63	1.69

Supplementary Table 2. Detailed outcome from the Bayesian model used in the Experiment 1, testing the effect of the SNR and the target position on the behavioral motivation.

	Co-located		Separated	
Behavioral scores	Highest probability	SNR	Highest probability	SNR
0	93.67	-20	69.68	-20
1	16.54	-14	16.47	-17.5
2	32.6	-11.8	32.87	-15.3
3	13.78	-9.8	14.09	-13.3
4	96.37	-2	99.6	-2

Supplementary Table 3. For each emission condition (co-located or separated) the highest probability of displaying the corresponding behavioral score (0, 1, 2, 3 or 4) and the corresponding SNR for the results of the Experiment 1.

		Estimate	Est. Error	l-95%	u-95%	Odds
Experiment 1	Intercept	9.18	2.38	4.21	13.61	×
	SNR	-0.06	0.17	-0.41	0.27	0.94
	Target position	-0.88	1.59	-3.93	2.44	0.41
Experiment 2	Intercept	18.12	21.43	-24.74	60.59	×
	SNR	-1.31	1.06	-3.42	0.78	0.27
	Target position	-11.44	10.33	-32.28	8.74	0.00
Experiment 3	Intercept	14.79	6.52	1.74	27.79	×
	SNR	0.05	0.25	-0.43	0.58	1.05
	Target position	2.06	2.24	-2.13	6.72	7.85

Supplementary Table 4. Detailed outcome from the Bayesian model used in the Experiments 1, 2 and 3, testing the effect of the SNR and the target position on the reaction times.

	Estimate	Est.Error	l-95% CI	u-95% CI
Intercept[1]	-7.63	2.09	-12.01	-3.86
Intercept[2]	-7.16	2.05	-11.49	-3.44
Intercept[3]	-5.62	1.89	-9.62	-2.17
Intercept[4]	-5.07	1.85	-9.03	-1.69
SNR	0.49	0.11	0.29	0.74
Angle	0.10	0.06	-0.01	0.23

Supplementary Table 5. Detailed outcome from the Bayesian model used in the Experiment 1, testing the effect of the SNR and of the separation angle on the behavioral motivation.

**Tested crocodile →<br>SNR (dB) ↓**	**1**	**2**	**3**	**4**	**5**	**6**	**7**	**8**
**-33**					0 / 1			
**-32**				0 / 1	0 / 1	0 / 1		
**-31**								0 / 1
**-30**				1 / 0	0 / 1			
**-29**			0 / 1	0 / 1				
**-28**			0 / 2				1 / 0	
**-27**		1 / 0	1 / 0			0 / 1	0 / 1	
**-26**			1 / 0	1 / 1	0 / 1			
**-25**	0 / 1	1 / 0		0 / 1		0 / 1	0 / 1	
**-24**	0 / 2				1 / 0			
**-23**	0 / 2		1 / 1		1 / 0			0 / 1
**-22**	1 / 0				0 / 1	1 / 0	1 / 0	
**-21**		1 / 1	0 / 1	1 / 0	1 / 1	1 / 0	0 / 1	
**-20**		0 / 1			1 / 1			2 / 1
**-19**		1 / 1				0 / 1	0 / 1	
**-18**	3 / 0			1 / 0				0 / 1
**-17**				0 / 1		1 / 1		
**-16**	1 / 0	1 / 0		1 / 0				0 / 1
**-15**			0 / 1					0 / 1
**-14**								
**-13**							1 / 0	

Supplementary Table 6. Details of the trials performed during the experiment 2 (N = 8 young Nile crocodiles). Each cell reports the number of stimuli (crocodile calls) played in the "co-located" and in the separated condition (left and right numbers, respectively).

	Estimate	Est.Error	l-95%	u-95%	Odds
Intercept	4.27	1.63	1.26	7.72	×
SNR	0.23	0.08	0.10	0.39	1.26
Target position	1.57	0.64	0.4	2.9	4.81

Supplementary Table 7. Detailed outcome from the Bayesian model used in the Experiment 2, testing the effect of the SNR and the target position on the detection.

	SNR	Contrast	Fitted (median)	Difference
min	-33.5	Co-located	2.94	8.78 [1.29, 27.57]
		Separated	12.51	
median	-22.1	Co-located	28.65	35.69 [8.94, 59.80]
		Separated	65.40	
max	-13.3	Co-located	74.79	17.09 [3.19, 41.27]
		Separated	93.36	

Supplementary Table 8. Contrasts of detection probabilities between the co-located condition and the separated condition for each SNR extremum: median of posterior distribution (%) and 95% CI for the results of the Experiment 2.

	Estimate	Est. Error	l-95%	u-95%	Odds
Intercept	7.05	2.64	2.71	12.98	×
SNR	0.26	0.11	0.08	0.51	1.30
Angle	-0.01	0.02	-0.04	0.03	0.99

Supplementary Table 9. Detailed outcome from the Bayesian model used in the Experiment 2, testing the effect of the SNR and of the separation angle on the detection.

Tested crocodile → SNR (dB) ↓	1	2
-32	0 / 2	1 / 0
-30	0 / 2	1 / 1
-28	0 / 5	2 / 4
-26	2 / 2	0 / 4
-24	4 / 4	1 / 8
-22	4 / 4	3 / 12
-20	6 / 6	4 / 7
-18	4 / 3	5 / 4
-16	4 / 2	3 / 4
-14	0 / 0	0 / 0
-12	0 / 1	0 / 1

Supplementary Table 10. Summary of all testing conditions for each individual considered in the experiment 3 (i.e. two conditioned juvenile Nile crocodiles). In each cell of the table, the left and right numbers correspond respectively to the number of target signals played in the co-located and in the separated condition.

	Estimate	Est.Error	l-95%	u-95%	Odds
Intercept	4.42	2.40	-0.85	9.54	×
SNR	0.20	0.05	0.1	0.32	1.22
Target position	0.58	0.44	-0.28	1.47	1.79

Supplementary Table 11. Detailed outcome from the Bayesian model used in the Experiment 3, testing the effect of the SNR and the target position on the detection.

	SNR	Contrast	Fitted (median)	Difference
min	-32	Co-located	11.67	6.11 [-3.89, 21.39]
		Separated	18.89	
median	-22	Co-located	49.36	12.45 [-5.70, 32.74]
		Separated	63.32	
max	-12	Co-located	87.63	4.28 [-2.51, 19.33]
		Separated	92.58	

Supplementary Table 12. Contrasts of detection probabilities between the co-located condition and the separated condition for each SNR extremum: median of posterior distribution (%) and 95% CI for the results of the Experiment 3.

	Estimate	Est. Error	l-95%	u-95%	Odds
Intercept	4.23	3.5	-2.81	11.66	×
SNR	0.17	0.07	0.05	0.31	1.19
Angle	0	0.01	-0.01	0.01	1.00

Supplementary Table 13. Detailed outcome from the Bayesian model used in the Experiment 3, testing the effect of the SNR and of the separation angle on the detection.

Exp.	Tested crocodile	Weight (kg)	Body length (cm)	Interaural distance (cm)
2	1	×	35.0	1.9
2	2	×	33.0	2.0
2	3	×	37.0	2.0
2	4	×	35.0	2.3
2	5	×	35.5	1.9
2	6	×	36.0	1.9
2	7	×	34.5	2.0
2	8	×	38.5	2.0
3	1	2.77	80.0	×
3	2	2.80	83.0	×

Supplementary Table 14. Biometric data of the Nile crocodiles (*Crocodylus niloticus*) considered in the Experiments 2 and 3.

731 ## Supplementary Movies

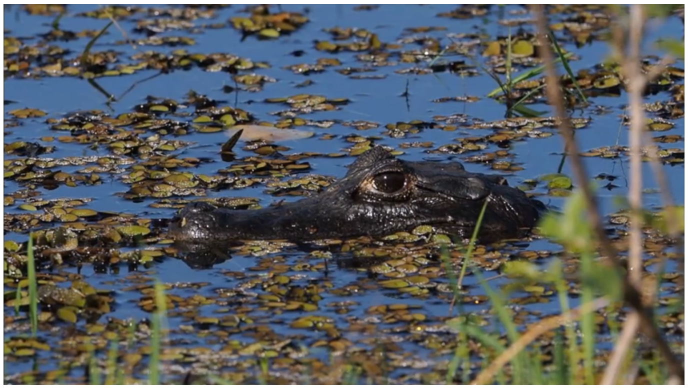

Supplementary Movie 1. Video footage of a playback experiment performed in the field (Pantanal, Brazil). The target loudspeaker which emits the sound stimuli (young caiman's calls) is on the left, out of the screen, 16 meters away from the female caiman. The noise loudspeaker is at the same distance. The angle between both loudspeakers is 20°. As shown by the video, the female caiman responds to the sound stimuli by swimming towards the target loudspeaker, in spite of the background noise. The video footage can be downloaded here[67]: https://doi.org/10.5281/zenodo.6522056.

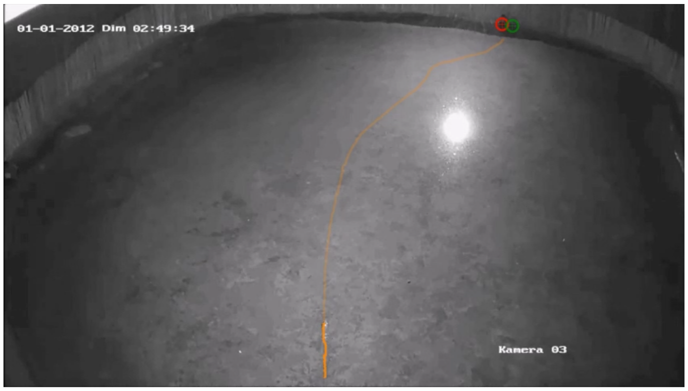

Supplementary Movie 2. Video footage of a playback experiment performed in captivity (Crocoparc zoo, Morocco). The noise loudspeaker (red circle) emits a continuous white noise. The target loudspeaker (green circle) emits the sound stimuli (young Nile crocodile's calls). As shown by the video, the tested Nile crocodile responds to the sound stimuli by swimming towards the target loudspeaker, in spite of the background noise. The video footage can be downloaded here[67]: https://doi.org/10.5281/zenodo.6522056.

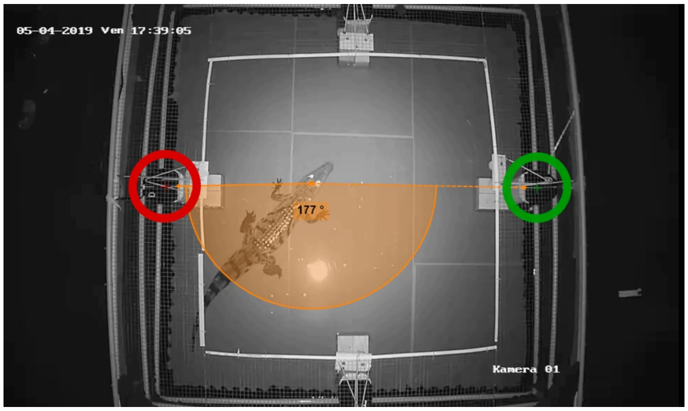

Supplementary Movie 3. Video footage of a Go/No-Go playback experiment performed in the laboratory. The target loudspeaker (green circle) emits the sound stimuli. The noise loudspeaker (red circle) emits a continuous white noise. Both loudspeakers are separated by an angle of 177°. As shown by the video, the crocodile responds to the sound stimuli by swimming towards the target loudspeaker, in spite of the background noise. The video footage can be downloaded here[67]: `https://doi.org/10.5281/zenodo.6522056`.
