# Supplementary material for: Spatial release from masking in crocodilians
Source: Commun Biol. 2022 Aug 25;5:869. doi: 10.1038/s42003-022-03799-7 (PMC9411511; doi:10.1038/s42003-022-03799-7)
Supplement: Supplementary file 3 — Description of Additional Supplementary Files [file 42003_2022_3799_MOESM3_ESM.pdf]

Description of Additional Supplementary Files

File name: Supplementary Movie 1
Description: Video footage of a playback experiment performed in the field (Pantanal, Brazil).

File name: Supplementary Movie 2
Description: Video footage of a playback experiment performed in captivity (Crocoparc zoo, Morocco).

File name: Supplementary Movie 3
Description: Video footage of a Go/No-Go playback exp.
