# Supplementary material for: Spatial release from masking in crocodilians
Source: Commun Biol. 2022 Aug 25;5:869. doi: 10.1038/s42003-022-03799-7 (PMC9411511; doi:10.1038/s42003-022-03799-7)
Supplement: Supplementary file 7 — Reporting Summary [file 42003_2022_3799_MOESM7_ESM.pdf]

nature portfolio

Corresponding author(s): Julie Thévenet & Léo Papet

Last updated by author(s): Jun 30, 2022

# Reporting Summary

Nature Portfolio wishes to improve the reproducibility of the work that we publish. This form provides structure for consistency and transparency in reporting. For further information on Nature Portfolio policies, see our Editorial Policies and the Editorial Policy Checklist.

## Statistics

For all statistical analyses, confirm that the following items are present in the figure legend, table legend, main text, or Methods section.

| n/a | Confirmed | |
|---|---|---|
| ☐ | ☒ | The exact sample size (*n*) for each experimental group/condition, given as a discrete number and unit of measurement |
| ☐ | ☒ | A statement on whether measurements were taken from distinct samples or whether the same sample was measured repeatedly |
| ☐ | ☒ | The statistical test(s) used AND whether they are one- or two-sided<br>*Only common tests should be described solely by name; describe more complex techniques in the Methods section.* |
| ☐ | ☒ | A description of all covariates tested |
| ☐ | ☒ | A description of any assumptions or corrections, such as tests of normality and adjustment for multiple comparisons |
| ☐ | ☒ | A full description of the statistical parameters including central tendency (e.g. means) or other basic estimates (e.g. regression coefficient) AND variation (e.g. standard deviation) or associated estimates of uncertainty (e.g. confidence intervals) |
| ☒ | ☐ | For null hypothesis testing, the test statistic (e.g. *F*, *t*, *r*) with confidence intervals, effect sizes, degrees of freedom and *P* value noted<br>*Give P values as exact values whenever suitable.* |
| ☐ | ☒ | For Bayesian analysis, information on the choice of priors and Markov chain Monte Carlo settings |
| ☒ | ☐ | For hierarchical and complex designs, identification of the appropriate level for tests and full reporting of outcomes |
| ☒ | ☐ | Estimates of effect sizes (e.g. Cohen's *d*, Pearson's *r*), indicating how they were calculated |

*Our web collection on statistics for biologists contains articles on many of the points above.*

## Software and code

Policy information about availability of computer code

| | |
|---|---|
| Data collection | All experiments were video-taped. The videos were analyzed using Kinovea software (https://www.kinovea.org/), an open source and free motion analysis software (Reference: Puig-Diví A, Escalona-Marfil C, Padullés-Riu JM, Busquets A, Padullés-Chando X, Marcos-Ruiz D. Validity and reliability of the Kinovea program in obtaining angles and distances using coordinates in 4 perspectives., 2019) |
| Data analysis | Statistical analyses were performed using R (v.3.6.2) - package "brms" (Reference: Bürkner, P.-C. brms: An R Package for Bayesian Multilevel Models Using Stan, 2017). |

For manuscripts utilizing custom algorithms or software that are central to the research but not yet described in published literature, software must be made available to editors and reviewers. We strongly encourage code deposition in a community repository (e.g. GitHub). See the Nature Portfolio guidelines for submitting code & software for further information.

## Data

Policy information about availability of data

All manuscripts must include a data availability statement. This statement should provide the following information, where applicable:

- Accession codes, unique identifiers, or web links for publicly available datasets
- A description of any restrictions on data availability
- For clinical datasets or third party data, please ensure that the statement adheres to our policy

Dataset, codes, videos, audio signals and supplementary information supporting the present results can be found in the Zenodo repository: https://doi.org/10.5281/zenodo.5971364

# Field-specific reporting

Please select the one below that is the best fit for your research. If you are not sure, read the appropriate sections before making your selection.

☒ Life sciences ☐ Behavioural & social sciences ☐ Ecological, evolutionary & environmental sciences

For a reference copy of the document with all sections, see nature.com/documents/nr-reporting-summary-flat.pdf

# Life sciences study design

All studies must disclose on these points even when the disclosure is negative.

| | |
|---|---|
| Sample size | Sample sizes were chosen to ensure sufficient precision of estimates of population-level effect sizes in Bayesian models. |
| Data exclusions | One experimental session was excluded in the third experiment, following the exclusion criteria that if the crocodile did not respond more than twice to one of the higher SNRs (-16 dB or -18 dB) in the same session, the entire session was excluded from the final data set, considering that the motivation to respond to the target signal was not sufficient. |
| Replication | Each experiment was replicated over a number of individuals. These number are stated in the manuscript for each experiment. |
| Randomization | Signals were played back at different intensity in a random order except for the first experiment, where the intensity of the calls was progressively increased until the first noticeable response of the caiman (due to experimental constraint once the animal has moved). This is specified in the manuscript. |
| Blinding | Video analysis of the behavioral responses was done by a person blind to the stimulus. |

# Reporting for specific materials, systems and methods

We require information from authors about some types of materials, experimental systems and methods used in many studies. Here, indicate whether each material, system or method listed is relevant to your study. If you are not sure if a list item applies to your research, read the appropriate section before selecting a response.

## Materials & experimental systems

| n/a | Involved in the study | |
|---|---|---|
| ☒ | ☐ | Antibodies |
| ☒ | ☐ | Eukaryotic cell lines |
| ☒ | ☐ | Palaeontology and archaeology |
| ☐ | ☒ | Animals and other organisms |
| ☒ | ☐ | Human research participants |
| ☒ | ☐ | Clinical data |
| ☒ | ☐ | Dual use research of concern |

## Methods

| n/a | Involved in the study | |
|---|---|---|
| ☒ | ☐ | ChIP-seq |
| ☒ | ☐ | Flow cytometry |
| ☒ | ☐ | MRI-based neuroimaging |

## Animals and other organisms

Policy information about studies involving animals; ARRIVE guidelines recommended for reporting animal research

| | |
|---|---|
| Laboratory animals | Nile crocodiles (Crocodylus niloticus), 3 years of age, born in captivity at the zoo ”La Ferme aux Crocodiles” and housed at the ENES animal facilities. |
| Wild animals | Morocco experiments: Nile crocodiles, 3 months of age, born in captivity in the CrocoParc. Animals were caught by the animal keeper to be placed in the experimental arena.<br>Brazil experiments: Jacare caiman (Caiman yacare), reproductive females. |
| Field-collected samples | The study did not involve samples collected from the field. |
| Ethics oversight | All experiments were performed in accordance with relevant guidelines and regulations including French and Brazilian national guidelines, permits and regulations regarding animal care and experimental use (approval no. D42-218-0901, ENES lab agreement, Direction Départementale de la Protection des Populations, Préfecture du Rhône). |

Note that full information on the approval of the study protocol must also be provided in the manuscript.
